# A life less lonely: the state of the art in interventions to reduce loneliness in people with mental health problems

**DOI:** 10.1007/s00127-017-1392-y

**Published:** 2017-05-20

**Authors:** Farhana Mann, Jessica K. Bone, Brynmor Lloyd-Evans, Johanna Frerichs, Vanessa Pinfold, Ruimin Ma, Jingyi Wang, Sonia Johnson

**Affiliations:** 1 Division of Psychiatry, University College London, 6th Floor, Maple House, 149 Tottenham Court Road, London, W1T 7BN UK; 2 McPin Foundation, 32-36 Loman Street, London, SE1 0EH UK; 3 Camden and Islington NHS Foundation Trust, St Pancras Hospital, 4 St Pancras Way, London, NW1 0PE UK

**Keywords:** Loneliness, Intervention, Social isolation, Social networks, Social prescribing

## Abstract

**Purpose:**

There is growing evidence of significant harmful effects of loneliness. Relatively little work has focused on how best to reduce loneliness in people with mental health problems. We aim to present an overview of the current state of the art in loneliness interventions in people with mental health problems, identify relevant challenges, and highlight priorities for future research and implementation.

**Methods:**

A scoping review of the published and grey literature was conducted, as well as discussions with relevant experts, to propose a broad classification system for types of interventions targeting loneliness.

**Results:**

We categorised interventions as ‘direct’, targeting loneliness and related concepts in social relationships, and ‘indirect’ broader approaches to well-being that may impact on loneliness. We describe four broad groups of direct interventions: changing cognitions; social skills training and psychoeducation; supported socialisation or having a ‘socially-focused supporter’; and ‘wider community approaches’. The most promising emerging evidence appears to be in ‘changing cognitions’, but, as yet, no approaches have a robust evidence base. Challenges include who is best placed to offer the intervention, how to test such complex interventions, and the stigma surrounding loneliness.

**Conclusions:**

Development of clearly defined loneliness interventions, high-quality trials of effectiveness, and identifying which approaches work best for whom is required. Promising future approaches may include wider community initiatives and social prescribing. It is important to place loneliness and social relationships high on the wider public mental health and research agenda.

## Introduction

Loneliness refers to a subjective unpleasant feeling arising from a mismatch between a person’s desired level of meaningful social relationships, and what they perceive they actually have [1]. It is related to (but distinct from) a range of concepts, such as social isolation, social capital, and social network, which are measured using objective means (Table 1). Crucially, it is the persistent subjective feeling of loneliness that has been shown to be a strong independent indicator of multiple physiological changes and poor health outcomes [2–8].

**Table 1:** Loneliness and related concepts in social relationships

Concept	Definition
Alienation	A feeling of disconnectedness from social settings such that the individual views his/her relationships from social contexts as no longer tenable^a^
Loneliness	A state of negative affectivity accompanying the perception that one’s social needs are not being met by the quantity or especially the quality of one’s social relationships^b^
Social capital	A series of resources that individuals earn as a result of their membership in social networks, and the features of those networks that facilitate individual or collective actions^c–e^
Bonding	Type of social capital. Closer connections between people with a family connection or shared group identity. Typically, the source of most of someone’s emotional and instrumental social support^f^
Bridging	Type of social capital. More distant connections between people not directly linked to friends or family, with distinctions or distance between them^f^
Social identity	Aligning one’s interests, attitudes, and behaviours with the groups to which one belongs but seeing them as different to those of groups to which one does not belong^g^
Social isolation	The inadequate quality and quantity of social relations with other people at the different levels, where human interaction takes place (individual, group, community, and the larger social environment)^h^
Social network	A specific set of linkages among a defined set of persons, with the additional property that the characteristics of these linkages as a whole may be used to interpret the social behaviour of the persons involved^i^
Perceived social support	Beliefs about the quantity and quality of support that is potentially available from the individual’s relationships and social contacts.^j,k^ This is distinct from received social support which is how often an individual reports receiving particular supportive behaviours from social contacts^j,k^

^a^Bronfenbrenner [96]

^b^Peplau and Perlman [97]

^c^McKenzie et al. [98]

^d^Portes [99]

^e^Putnam [100]

^f^Veronique [101]

^g^Turner and Oakes [102]

^h^Zavaleta et al. [103]

^i^Mitchell [104]

^j^Dour et al. [105]

^k^Hupcey [106]

Transient loneliness may have an adaptive purpose [9], driving the individual to find ways to reduce it, but prolonged loneliness is increasingly recognised as a significant public health issue [10, 11]. Over a third of over-50s report experiencing loneliness in the UK [12], and one in five US adults, with evidence the prevalence may be increasing [13]. There is also a peak in adolescence [14].

Much of the existing work on loneliness and its impact on health have been conducted with older adults, demonstrating both increased morbidity and mortality [15–17]. In comparison, relatively little work has focused on the impact of loneliness in people with mental health problems, and consequently how best to tackle it.

Marked cross-sectional associations exist between loneliness and mental health problems [18], including depression (OR 10.85) and anxiety (OR 11.56) [19]. People who feel lonely are at increased risk of Alzheimer’s disease and cognitive impairment [7, 20, 21]. Loneliness is also correlated with eating disorders [22], sleep problems [23], and both suicidal ideation and suicide attempts [24]. Fewer studies look at its relationship with psychosis, but one survey showed 80% of people reported it as a ‘major challenge’ with levels significantly higher than in the general population [25], while another highlighted loneliness in ultra-high-risk-for-psychosis states [26].

The UK National Health Service (NHS) Five-Year-Forward View for Mental Health [27] mentions peoples’ wish for good quality relationships, but does not discuss the impact of loneliness. We argue that there is an urgent need to consider how best to address this issue. This comes in the context of a renewed drive to consider social determinants of mental health outcomes [28].

## Aim

We aim to present an overview of the current ‘state of the art’ in interventions to address loneliness (and closely related constructs) in people with mental health problems. This has been achieved through a scoping review of the literature as well as discussions with relevant experts, including academics, clinicians, service users, and social entrepreneurs. All the interventions included have the potential to alleviate loneliness, although we have included studies with varying primary targets (i.e., loneliness, social isolation, social networks, and social support), given the potential relevance to future loneliness interventions. We draw on the available evidence and emerging themes to describe existing approaches and challenges, and propose future priorities in the complex field of improving social relationships and mental health.

## Existing reviews

Early reviews on tackling social isolation in older people highlighted the value of social skills training and suggested educational groups were most effective, while others concluded that there was little evidence for effective interventions at all [29, 30].

A 2011 meta-analysis of loneliness and social isolation interventions [31] identified four primary strategies: (1) improving social skills; (2) enhancing existing social support; (3) increasing opportunities for new social contact; and (4) addressing maladaptive social cognitions. Only one small study included people with ‘serious and persistent’ mental health problems. Compared with other designs in the meta-analysis, the 20 randomised controlled trials (RCTs) showed the smallest effect size (−0.198). Of the four intervention strategies, the four RCTs of cognitive interventions yielded the greatest effect size (−0.598) compared with, for example, 12 studies enhancing social support (−0.162). This sits well alongside evidence of cognitive changes in people who feel lonely [32], but the small effect sizes and few studies of people with mental health problems included limit generalisation of these findings.

A more recent systematic review looked at interventions targeting social networks (not loneliness) in psychosis, and found five trials of highly varied interventions, with some limited evidence of effectiveness [33].

## A classification system

Our literature search and discussions with relevant experts contributed to a proposed classification system (Table 2). We refer to interventions explicitly targeting loneliness and related concepts in social relationships as ‘direct’ interventions. In contrast, ‘indirect’ broader approaches to health and well-being do not specifically aim to address loneliness but nevertheless may have important impacts on loneliness. Under direct interventions, we describe four groups: ‘changing cognitions’, ‘social skills and psychoeducation’, ‘supported socialisation’, and ‘wider community approaches’. The interventions fitted broadly into these categories based on their main approach, but they are not mutually exclusive. We describe the interventions, along with relevant evidence where available, in more detail below.

**Table 2:** Classification system for types of intervention directly targeting loneliness

Category	Description	Modes of delivery (examples)
Changing cognitions	Interventions that aim to reduce ‘maladaptive’ cognitions in lonely people. They may target cognitive biases or attributional styles, changing the way individuals think about their social relationships. This aims to change behaviours, increasing social connections and decreasing loneliness	Through mental health services School-based Individual sessions Group sessions Digital interventions
Social skills training and psychoeducation	Interventions that focus on training in or education on one’s social skills, such as conversational ability and interpreting body language. Psychoeducation may focus on managing mental health problems alongside the importance of social support. This aims to enable individuals to form and maintain meaningful relationships and thus reduce loneliness	Through mental health services Individuals or families Group sessions Digital interventions Using peer support
Supported socialisation or having a ‘socially-focused supporter’	Interventions where people are offered support and guidance in finding and attending new activities or groups. A specific supporter (a professional, family member, friend, volunteer or peer supporter) works towards social goals with the lonely person. They aim to help individuals make social connections which can be maintained after their support ends, thereby reducing loneliness	Individual support, provided by Mental health services Charity and third sector organisations Local community Peer supporters Working with primary care
Wider community groups	Interventions include groups that appeal to a wider range of members, with or without mental health problems. These aim to facilitate better integration into the community, reduce stigma and boost the lonely person’s confidence as a member of a wider society which is receptive to them	Groups, facilitated by Local community organisations Charity and third sector organisations Working with primary care

These broad groups are not designed to be mutually exclusive, and there is scope for approaches to be combined in some interventions. Some examples of modes of delivery being used are given

## Changing cognitions

These interventions aim to shift the so-called ‘maladaptive’ cognitions in people experiencing loneliness. There is evidence people who feel lonely (whether they have mental health problems or not) have particular cognitive biases and attributional styles, which may overlap with cognitions contributing to mental health problems. Loneliness can lead to negative evaluations of other people, for example, and a lack of interpersonal trust [5]. Different cognitive mechanisms may contribute to maintaining loneliness at different stages of life [34] and recent studies highlight the potential for negative schemata in leading to paranoia in psychosis [26, 35].

The intended mechanism is changing an individual’s thinking, for example, the way they think about themselves in relationships, their possible assumptions about other peoples’ views, or their expectations of ‘success’ at overcoming loneliness. The hypothesis is that these changes can in turn lead to changed social behaviours and a reduction in individual loneliness over time. Such interventions can be delivered on a one-to-one basis, in groups or through digital technology.

As part of an ongoing related systematic review, we identified ten published RCTs on cognitive approaches to improving loneliness or related concepts (social support/social networks/social isolation) in people with mental health problems [36–45]. In depression, ‘online cognitive behavioural therapy (CBT) plus motivational interviewing (MI)’ was compared with ‘online CBT plus brief advice’ in primary care. Both interventions reduced loneliness at 12 months, with no advantage in adding the MI component [37]. Another study tested whether cognitive ‘reframing’ of loneliness led to greater perceived control over reducing it, but found no significant impact [36].

In mothers with depression postpartum, neither ‘specialised CBT’ nor ‘internet-based behavioural activation’ had a significant impact on perceived social support [40], and an older study showed no benefit in adding ‘cognitive modification’ in social phobia [44]. In children experiencing grief and trauma, there was no added benefit from narrative therapy on social support [38]. Two small, related, trials of narrative exposure therapy in post-traumatic stress disorder provided mixed results: one showed an improvement in social support, while the other did not [43, 45].

Considering the general population, a recent integrative review concluded that a range of approaches to reduce loneliness and social isolation in older people are currently being tested or developed. It identified ‘psychological therapies’ as having the most ‘robust’ evidence to date. However, all the interventions were delivered in groups, and it was not possible to determine the relative contributions of general group interactions compared with the specific therapeutic intervention [46]. In younger people, a recent feasibility trial of a mindfulness intervention in Chinese college students suggested that it could reduce loneliness scores [47].

A key consideration in interventions of this type is that the people offered treatment have definite cognitive biases, which in turn impact on their loneliness. However, none of the studies identified take steps to specifically measure such biases at baseline. Targeting a mixed group of lonely people, who may or may not demonstrate such cognitive patterns, could mean important treatment effects are missed.

All in all, the evidence base for cognitive interventions for loneliness is in its infancy, despite being the best studied of the intervention types described here. Larger, adequately powered studies with longer follow-ups, and loneliness as a primary outcome, will be more informative. Future studies should explore the mechanisms involved in bringing about any changes and how delivery mode (e.g., digital, group, or individual) influences effectiveness.

## Social skills training and psychoeducation

These interventions focus on practical ‘training’, education, or improving awareness of ‘social skills’, to reduce loneliness or improve social support. ‘Skills’ include a broad range, such as conversational ability and reflecting on body language. Psychoeducational programmes may be aimed at individuals, groups, and/or families, are typically diagnosis-focused, and include information on the importance of social relationships. The hypothesis is that such practical advice and information will better equip the individual to form meaningful relationships and have better skills to prioritise and maintain them over time. There is of course scope for such interventions to be combined with psychological therapy approaches, such as CBT.

RCTs of social skills/psychoeducation have been conducted with people with bipolar disorder or schizophrenia, but all measured perceived social support, and not loneliness. An online self-help intervention, including psychoeducation and group discussion boards, led to a significant improvement in social support [48], though the study had a high attrition rate. Four further RCTs did not show significant changes in social support after psychoeducation programmes, including a mixed diagnosis peer-delivered recovery course [49–52].

A social skills, psychoeducation, and physical health group in older people with depression had no impact on social support [53]. A recent review of social skill groups to reduce loneliness in high-functioning autism concluded that there was ‘tentative’ evidence, they are effective, but the studies included were either quasi-experimental or single-arm trials [54].

Dr. Catherine Haslam, clinical psychologist and academic, discussed the ‘Groups 4 Health’ project (interview with JB), which takes a ‘social identity’ approach. It features modules educating people about the importance of social relationships and strengthening relevant resources. There was a notable dropout rate, but in students with mood disturbance, there was a reduction in loneliness [55]. Social identity theory essentially posits that people derive an important part of their identity and self-esteem through belonging to groups (e.g., being ‘American’/being a football fan). The hypothesis is that such a sense of social identity is a key mechanism in why groups (such as psychoeducation or indeed psychological therapy groups) may have beneficial effects on mental health [56].

Of note, a recent systematic review found that active therapy groups were no more effective than sham therapy groups in schizophrenia [57]. An important consideration in all group interventions addressing loneliness will, therefore, be to take steps to consider group interaction effects.

It is possible that social skill programmes may work best in combination with other approaches, or may be suited to people who prefer not to engage with in-depth psychological therapy or wish for ‘preparation’ before attending wider community groups. There is scope for such interventions to be delivered to individuals, groups, by peers or digitally. The evidence of an impact on loneliness, however, is at this point limited.

## Supported socialisation or having a ‘socially-focused supporter’

Here, people are offered support and guidance to select and attend activities. There is some overlap with ‘social skills’, but in these interventions, there is a particular person assigned to help the individual work towards specific goals (usually attendance at groups to reduce loneliness). The support may be from professionals, family, other volunteers, or peer support workers and is typically (though not necessarily) time-limited. The ‘supporter’ aims to improve the chances of successfully reducing loneliness by supporting the person to make their own decisions, jointly reviewing their needs, identifying what support might be helpful, helping select the most appropriate activities/groups, and providing relevant motivation and guidance.

An RCT of ‘supported socialisation’ involved volunteers identifying suitable community activities for an individual, and supporting the person to attend for 3–6 months [58]. They found social network improvement but did not measure loneliness and the improvement was not found for the people classed most clinically unwell.

An RCT including people with severe mental health problems compared matching with a volunteer plus a stipend (20 Euros), against offering a stipend alone, to promote group attendance [59]. Both conditions led to an improvement in social loneliness with no additional benefit from having the volunteer. The authors comment: ‘…it is possible the demand to establish a ‘friendship’ with a stranger within the study timeframe…mitigated against realising the benefits of such friendships in the short term.’ A previous review suggested that the majority of benefits accrued through befriending programmes tend to occur after the first year [60]. Whether provision of a stipend alone (compared with treatment as usual) provides additional benefits may be worth exploring in the future.

Formal peer support (PS) refers to organised support provided by people with lived experience of mental health problems (as opposed to naturally occurring informal support). PS trials identified were relatively new and conducted in mixed diagnostic groups [61–63], but had no significant impact on loneliness.

A systematic review on PS interventions (without a specific focus on loneliness) concluded that there was limited evidence on their effectiveness in severe mental illness, and suggested that new interventions are best rolled out in the context of well-designed research studies [64]. One challenge is accounting for the wide variation in what peers deliver, and demonstrating that it is indeed the active peer support element that is driving change. RCT data from an ongoing UK study trialling a PS intervention in people with mental health problems are expected in the near future [65], including assessing its impact on loneliness.

Mark Swift (social entrepreneur at Well-being Enterprises) [66] discussed employing ‘well-being officers’ who offer one-to-one support around well-being, including loneliness. Such ‘well-being officers’ offer particular knowledge about their local communities, and insights into what specific opportunities may be available for the individual they see, as well as advice on how to develop new groups or activities in the community. The ‘Hounslow Well-being Network’ [67] builds on ‘network-mapping’ research and offers ‘asset mapping’ to help people better understand their social networks [68].

A feasibility trial employing ‘community navigators’ to work with clients in secondary mental healthcare is currently under way [69]. The ‘navigator’s role is to collaboratively map out and review an individual’s existing relationships (including any strengths), develop an ‘action plan’ to engage in any activities identified, and offer practical and financial supports. Such link workers are an important part of social prescribing (discussed below).

None of the identified studies explored any role for personality features, e.g., introvertedness, and how these influence ‘responsiveness’ to such interventions. Baseline measures of such characteristics may give more informative results.

The ‘Campaign to End Loneliness’, in a recent report, discussed the importance of one-to-one support for people for whom the barriers to ‘getting out’ were too great [70]. In the general population, they point out that the evidence is ‘too weak’ to state that such initiatives (though ‘highly valued’) are effective at reducing loneliness.

## Wider community groups

Given the apparent modest impact of cognitive approaches or social skills training, one argument is that they do not take into account the wider context in which the individual exists. Targeting an individual’s cognitions and preparing them to ‘get involved’ in their community may have limited impact if there are no efforts to create a broader sense of connectedness in the community itself. Groups that appeal to a wider range of members, with or without mental health problems, may facilitate better integration, reduce stigma, and boost one’s confidence as a member of wider society.

### Social prescribing (SP)

There is currently no widely agreed definition of SP with the 2016 SP Annual Report highlighting 56 different variations [71]. In essence, SP can refer to either: a) the process of healthcare professionals (e.g., a general practitioner) prescribing time with a link worker (e.g., a community navigator) or b) both the process of prescribing a link worker and the subsequent community group/activity that is recommended to the service user. SP establishes a link between health services and potentially a very broad range of social interventions, with the aim of improving health and well-being (“Box 1”). With an estimated 76% of family doctors reporting between one and five patients a day attend primarily due to loneliness (whether or not they identify it as the reason themselves), primary care is one important point at which to identify lonely people [70].

The UK government has previously called for the development of SP approaches in managing ‘chronic health problems’. However, a 2015 report on SP [72] found little evidence to support widespread commissioning, making this a research priority.

### Asset-based community development (ABCD)

Several experts interviewed and discussed the importance of involving various groups within communities in identifying and mobilising individual and community ‘assets’, as opposed to only focusing on deficits. ABCD supports and encourages people to develop their own community projects, with the aim of improving both relevance of the groups to local individuals and sustainability (e.g., [73]).

People who feel lonely may, therefore, be identified by a family doctor and prescribed a social intervention. This in turn comprises time with a person exploring their social networks and circumstances, and referring on to community groups that have developed as a result of ABCD, or encouraging the individual to set up their own group (promoting social entrepreneurship). Numerous promising ABCD initiatives currently exist, but, as far as we are aware, none have been subjected to peer-reviewed research as yet.

### Promoting city-wide loneliness initiatives

In 2016, ‘Macc’ (a voluntary sector organisation) summarised the results of a series of projects aimed at reducing loneliness and social isolation across the UK city of Manchester [74]. Various organisations ran groups ranging from befriending to communal eating and psychological therapy, taking referrals from health and social services and primary care. Again, there is no peer-reviewed evidence published, but their own evaluation suggested improvements in self-reported health and well-being. Such interventions may be a mix of direct and indirect approaches (e.g., a ‘loneliness’ psychology group versus a ‘managing diabetes’ group). Trials of such complex that varied community-wide interventions will be challenging to design and evaluate; we suggest that researchers draw inspiration from trials in other priority public health areas, such as dietary education [75].

The aim is to bring about much wider awareness and active participation in promoting social relationships and reducing (or preventing) loneliness. This in turn theoretically provides people experiencing loneliness and mental health problems with a more receptive environment within which to develop. This is a potentially valuable approach, given evidence that various social factors can influence the likelihood of experiencing loneliness.

Across these interventions, there is a pressing need to match the growing service report and non-peer-reviewed evidence on well-being, with high-quality research on effectiveness across outcomes, including loneliness. There is otherwise a risk of commissioning services that may not have any lasting impact on loneliness and related health outcomes.

## Indirect interventions

There are a potentially vast range of indirect interventions that may plausibly impact on loneliness. For example, loneliness is known to be associated with poverty [76], thus efforts to improve inequalities may have a significant impact on loneliness levels. Wider initiatives to improve employment opportunities (e.g., individualised placement and support), education (e.g., recovery colleges), or housing may also be highly relevant. Initiatives that bring people together for other purposes, such as physical health programmes, may also be valid approaches to reducing loneliness. Consideration of loneliness as an outcome in these and other relevant policies/interventions should be a priority.

## Primary prevention

It is important to place loneliness higher on the public mental health agenda. A key component of this could be raising public awareness of the value of healthy social relationships, similar to campaigns for other aspects of healthy living, such as eating well or exercise. Communities that are better informed may be more likely to actively engage in supporting those at risk of prolonged loneliness.

In an interview for this paper (with FM), Professor de Jong Gierveld discussed her concern that the modest impact of loneliness interventions to date may reflect the need to focus on intervening much earlier, preventing more chronic or ‘harder to shift’ loneliness from becoming established. She highlighted the need to educate people about actively investing in their ‘social convoys’ (the range of existing social bonds in their life, such as family and friends), but also pointed out the critical importance of boosting the individual’s own motivation to actively change their situation [77].

The NHS Five-Year-Forward View [27] calls for a focus on prevention in important ‘lifestyle’ areas, such as obesity, but not specifically social relationships. Similarly, the UK National Institute for Clinical Excellence (NICE) guidance on, e.g., schizophrenia or depression, do not currently promote social relationships as areas to focus on assessments. There has, however, been recent NICE guidance issued on managing loneliness in the elderly [78].

## Challenges

### Who is lonely?

In older people, methods, such as using ‘geographical information systems’, aim at identifying vulnerable people [79]. In the UK, older people who have frequent hospital attendances are identified as vulnerable to isolation. In the mental health setting, the ‘Connecting People’ Study showed that people using secondary mental health services had better access to social capital than those only accessing voluntary services [80], but we lack similar information in people with mental health problems who experience loneliness.

### Stigma

Experts interviewed for this paper discussed how people find it difficult to talk about loneliness, including the fact that they may under-report it in questionnaires. Public education initiatives would be one way of trying to reduce negative or shameful attitudes to being lonely. There is then the added stigma of mental illness, both from others and internalised.

Dr. Louise Hawkley (psychologist and senior researcher) emphasized the societal barrier to inclusion as being one of the biggest hurdles faced by people with mental health problems (interview with FM). Addressing issues of social exclusion and marginalization of course require consideration of wider societal issues, including where people with mental health problems are living or employment opportunities.

### What about the measures?

The two most commonly used loneliness scales are the UCLA Loneliness Scale [81] and the de Jong Gierveld loneliness scale [82]. Both were initially developed in the general population, but have been used widely in mental health studies. Two recent reviews explored a wide range of tools used to measure social relationships [83, 84], highlighting overlap between ‘objective’ and ‘subjective’ scales. Having reliable and valid measures and clear definitions is fundamental to good quality research. Yet, when trying to measure a concept as individual and nuanced as feeling ‘lonely’, they can only go so far.

The most detailed means of unravelling the experience is sound qualitative research and using the findings to sit alongside and inform quantitative work. This should extend to qualitative work exploring the acceptability of current loneliness scales and developing more acceptable tools if necessary. In several interviews, including with academic psychiatrist Dr. Domenico Giacco, we discussed the difficulty of measuring the more ‘abstract’ concept of loneliness and questioned whether more tangible concepts, such as social networks, present a more practical target. Another challenge is ascertaining how loneliness relates to other meaningful improvements in people’s lives, including clinical outcomes, and trajectories of loneliness over time.

### One size does not fit all

There is a need to consider the needs of less well-researched groups, such as people with physical disabilities and people that typically get excluded from large trials (e.g., substance misuse populations [85] or different cultural groups [86]. One way of accounting for such differences is to explore each person’s needs early on in any intervention. Mapping an individual’s ‘well-being network’ is one way of having a conversation about existing networks and activities, as well as their impact on well-being. Mapping allows people to explore whether certain relationships are harmful to well-being, as well as identifying where changes could be made. The process has been evaluated positively in qualitative interviews [87]. The ‘Connecting People’ study used ethnographic methods to explore how professionals and people with psychosis discussed social networks and developed a tool to support this process [80].

Any exploration of the complex feeling of loneliness should ideally explore the person’s individual circumstances, including their views on what may have ‘led’ to their feeling lonely. An intervention for a bereaved older man may be different to the approach chosen to help an adolescent struggling with social skills, for example.

### Whose problem is it anyway?

**Fig. 1:**
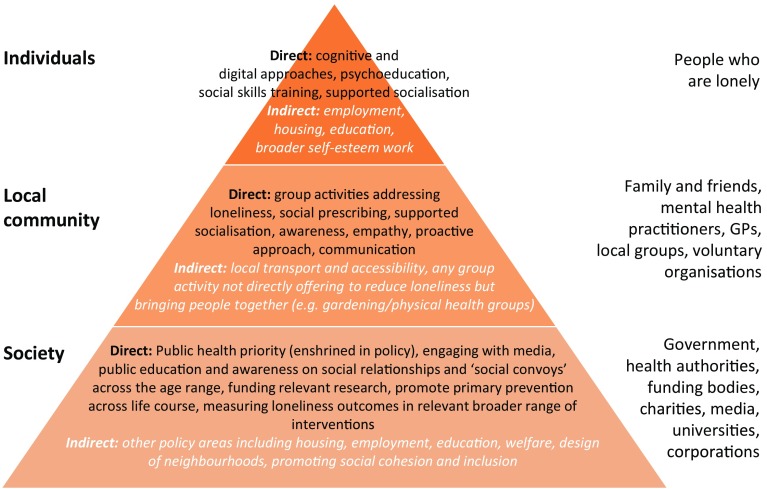
Levels of responsibility for interventions in loneliness. Examples of possible interventions for loneliness in people with mental health problems at each level. There is overlap and crossover between the levels, e.g., supported socialisation requires someone to help the individual socialise and groups and activities for the person to attend. Interventions at the community level may also require societal change

A key consideration is the issue of where various responsibilities lie. Professionals can find it hard to recognise where the boundaries between their role(s) and the individual’s own responsibilities begin in this context [87]. Thinking more broadly, there is the distribution of responsibilities between various levels in society (Fig. 1). With social prescribing projects potentially funded by health or social care, and provided by local authorities, third sector or health services, there is potentially no clear ‘ownership’ of such projects. Qualitative work with older people has highlighted some peoples’ reluctance to discuss loneliness in healthcare settings [88].

## Other research priorities

### Synthesizing evidence

Synthesizing existing evidence systematically, demonstrating effectiveness and subjecting it to peer-review, will strengthen arguments for investment and wider development of such approaches. This is best achieved through a combination of quantitative and qualitative work. It will require partnerships between third sector (non-statutory) or other organisations and academic institutions, each bringing their own expertise. A centralised database, dedicated to innovations or information-sharing on social relationship interventions, could be helpful.

### Which aspect(s) should we focus on?

It has been argued that social isolation is of greater relevance in predicting poor physical health [89], while others have argued that loneliness is a stronger predictor [8]. There is also evidence of the impact of social *capital* on mental health [90], as well as other aspects of social relationships. Future studies that tease out how such distinct but related factors interact will be crucial in improving our understanding of mechanisms involved.

### Missing mechanisms?

Further exploration of the mechanisms involved in leading to, or maintaining, loneliness will also be crucial. Most existing work has been in the general population, but one study suggests that loneliness mediates a relationship between internalised stigma and depression in people with psychosis, while another suggested that loneliness is a contextual moderator that may strengthen the trauma–psychosis relationship [91]. Other relevant psychological phenomena, such as motivation and level of perceived control/autonomy, were also highlighted as under-investigated. Dr. Hawkley pointed out trials that demonstrated loneliness interventions to be effective only when the participants had greater perceived control over various aspects of the intervention [92]. There is also a growing body of evidence for likely physiological impacts/correlates of loneliness [13].

## Conclusion

An impressively wide range of interventions to reduce loneliness and related constructs are already being run in various different communities. These typically involve older people, but some support adults with mental health problems. As yet, no types of intervention have a robust evidence base. Development of clearly defined interventions, high-quality trials of effectiveness, and exploration of which approaches might work best for whom is required. Promising future approaches may include: public health initiatives to create accepting communities, better designed psychological intervention studies, greater use of digital technology and programmes to link people with supportive social activities, and opportunities within local communities. All of these must be considered in the context of wider social policies, including housing, employment, welfare benefits, and infrastructure, to support forming meaningful social relationships that may improve health outcomes and quality of life for people with mental health problems.

## Box 1

Examples of well-established social prescribing programmes in the UK, although many more programmes are in existence across the UK and beyond. These programmes aim to improve health and well-being by linking people to a range of community-based activities and increasing social connections.

**Men’s Sheds** [93]

Having originated in Australia, Men’s Sheds are venues, where members share tools and resources to work on community projects. Sheds mostly attract older men, but anyone can join and some have included women and young people. Sheds facilitate skill-sharing, community projects, and social interaction.

**The Bromley by Bow Centre** [94]

A healthcare professional can refer any adult to a social prescribing coordinator. They discuss what is important for the person’s well-being, identify beneficial local activities and services, and support people to start using them. Services may include financial support, volunteering, arts, or support groups.

**The Rotherham Social Prescribing Service** [95]

People with long-term health problems can be referred to Social Prescribing Workers who identify suitable voluntary or community services. For those with poor mobility, transport or low confidence, workers will help them access services and activities to improve their health and well-being.

**Well-being Enterprises** [66]

Anyone can self-refer to a ‘Community Well-being Officer’ who performs a ‘well-being review’, including social connections and how to overcome challenges. Officers enrol people in activities, such as practical, well-being-focused, or creative courses, where they can meet others and learn new skills.
